# CSF and serum ferritin levels in narcolepsy type 1 comorbid with restless legs syndrome

**DOI:** 10.1002/acn3.51056

**Published:** 2020-05-20

**Authors:** Lucie Barateau, Sofiene Chenini, Manuela Lotierzo, Anna Laura Rassu, Elisa Evangelista, Régis Lopez, Anne‐Marie Gorce Dupuy, Isabelle Jaussent, Yves Dauvilliers

**Affiliations:** ^1^ Sleep‐Wake Disorders Unit Department of Neurology Gui‐de‐Chauliac Hospital CHU Montpellier Montpellier France; ^2^ National Reference Network for Narcolepsy CHU Montpellier Montpellier France; ^3^ Neuropsychiatry: Epidemiological and Clinical Research INSERM University Montpellier Montpellier France; ^4^ Department of Biochemistry Montpellier University Hospital Montpellier France; ^5^ PhyMedExp INSERM U1046 CNRS UMR 9214 University of Montpellier Montpellier France

## Abstract

**Objectives:**

To investigate whether cerebrospinal fluid (CSF) and serum ferritin levels differ between patients with narcolepsy type 1 (NT1) comorbid with restless legs syndrome (RLS) or periodic leg movements during sleep (PLMS), and patients with NT1 or controls without comorbid RLS or PLMS.

**Methods:**

Sixty‐six drug‐free patients with NT1 (44 males, age 38.5 years [14–81]) were enrolled, including 20 with RLS, 18 with PLMS index ≥15/h (six with both RLS and PLMS). Thirty‐eight drug‐free patients (12 males, age 22.5 years [12–61]) referred for sleepiness complaint, but without central hypersomnia, RLS, PLMS were included as controls. Clinical, electrophysiological and biological (CSF/serum ferritin, orexin [ORX]) data were quantified.

**Results:**

NT1 patients with and without RLS did not differ for age, gender, and body mass index (BMI). No between‐group differences were found for CSF ferritin, ORX, and serum ferritin levels. No CSF ferritin, ORX, and serum ferritin level differences were found between NT1 patients with and without PLMS, or with RLS or PLMS versus not. CSF‐ferritin levels were not different between NT1 and controls in adjusted analyses. CSF‐ferritin levels in the whole population correlated positively with age, serum‐ferritin, BMI, negatively with ORX, but not with PLMS index. In NT1, CSF‐ferritin levels correlated with age and serum‐ferritin but not with PLMS.

**Conclusion:**

The absence of CSF ferritin deficiency in NT1 with comorbid RLS or PLMS indicates normal brain iron levels in that condition. This result suggests that the frequent association between RLS, PLMS, and NT1 is not based on alterations in brain iron metabolism, a pathophysiological mechanism involved in primary RLS.

## Introduction

Narcolepsy type 1 (NT1), also called orexin (ORX)/hypocretin deficiency syndrome, is a rare sleep disease characterized by excessive daytime sleepiness, cataplexy, hypnagogic hallucinations, sleep paralysis, and disturbed nocturnal sleep.^1^ Conversely, restless legs syndrome (RLS) is a common sensorimotor disorder, characterized by an urge to move the legs accompanied by uncomfortable sensations, worsening at night.^2^ In European and American populations, 2–3% of adults suffer from clinically significant RLS symptoms. RLS has been initially categorized into primary (or idiopathic) and secondary (symptomatic) cases. However, recent studies support the idea that RLS might be seen as a continuous spectrum with a major genetic contribution at one end and a major environmental or comorbid disease contribution at the other.^3^, ^4^

Associations between NT1 and RLS have been reported in up to 14.7% of cases in a large case‐control study.^5^ However, unlike primary RLS, RLS in NT1 is not more prevalent in women, is less familial, and symptoms are often less severe. It has been thus hypothesized that pathophysiological mechanisms underlying RLS comorbid with NT1 could be different from primary RLS.^5^ The role of brain iron insufficiency and dopamine neurotransmission abnormalities, in genetically predisposed individuals, are now well‐established in the etiology of primary RLS.^6^ Three studies reported that ferritin levels in cerebrospinal fluid (CSF) of primary RLS patients are reduced, with correlations between CSF and sera ferritin levels being weaker in RLS than in controls.^7^, ^8^, ^9^ Conversely the nature of the association between RLS and NT1 remains unclear, and brain iron stores have never been explored in NT1 comorbid with RLS.

Periodic leg movements during sleep (PLMS) are repetitive, involuntary movements of lower limbs during sleep. PLMS are highly prevalent in RLS, and are also particularly frequent in NT1.^10^, ^11^ Electrophysiological features of PLMS in NT1 also differ from primary RLS, regarding their time structure, periodicity, and overnight distribution.^12^, ^13^ To the best of our knowledge, the relationship between CSF ferritin levels and PLMS has rarely been studied in RLS,^8^ and never in NT1.

In this study, we aimed to investigate whether CSF and serum ferritin levels differ between (1) NT1 patients with and without comorbid RLS, (2) NT1 patients with and without PLMS, and (3) NT1 patients with controls.

## Methods

### Population

Sixty‐six drug‐free patients with NT1 (44 males, median age 38.5 years [14–81]), diagnosed at the French National Reference Center for Narcolepsy in Montpellier were included. Among them, 35 were drug naïve and 31 in withdrawal condition for treatment of NT1. The diagnosis of NT1 was based on the third International Classification of Sleep Disorders criteria.^14^ All patients had a typical narcoleptic phenotype, with clear‐cut cataplexy and low ORX‐A CSF levels (<110 pg/mL). They all underwent a comprehensive clinical evaluation including body mass index (BMI), age at narcoleptic symptoms onset, frequency of cataplexy using the Cataplexy Frequency Scale,^15^ hypnagogic/hypnopompic hallucinations, sleep paralysis, parasomnias (REM‐sleep behavior disorder and non REM‐sleep parasomnia), and Epworth sleepiness scale (ESS) score. A specific attention was paid to assess the presence of comorbid RLS, diagnosed by medical sleep expert interview, based on the International Criteria,^2^ its severity (assessed clinically by sleep experts, with a clinical global impression CGI scale), age of onset, and positive family history. Only patients with NT1 with moderate to severe typical RLS (as assessed by CGI) and/or PLMS index ≥15/h were included. Other patients with NT1 (without RLS, and with PLMS <15/h) were chosen to be comparable for age, gender, and BMI to the first sample. NT1 patients who had rare, mild or doubtful RLS were not included in this study. The possibility of a drug induced RLS was systematically explored and ruled out, as some drugs used for narcolepsy may induce or worsen RLS (especially antidepressants used as anticataplectic drugs). All patients were drug‐free at time of evaluation: the medical interview for potential diagnosis of RLS, polysomnography (PSG) and lumbar puncture were performed during the same hospitalization.

Thirty‐eight drug‐free other patients (12 males, median age 22.5 years [12–61]) were referred for evaluation of hypersomnolence at the French National Reference Center for Narcolepsy in Montpellier, and underwent the same standardized evaluation, in drug‐free condition, with clinical, electrophysiological and biological assessment. They had no RLS, no objective daytime sleepiness with normal latencies on the multiple sleep latency tests (MSLT), no clinically significant polysomnographical abnormalities (PLMS index <15/h, apnea hypopnea index <15/h), and normal CSF ORX‐A levels (median levels 307 pg/mL [210–504]). Those patients complaint of hypersomnolence and may also experience mild sleep‐disordered breathing, disturbed nighttime sleep, sleep deprivation, or mild depression, and thus do not constitute a healthy control group. However, a diagnosis of central hypersomnolence disorder was ruled out, and they were named under the entity “non‐specified hypersomnolence.”

None of the participants had renal or liver failure, anemia, or comorbid inflammatory disease. None of participants have been previously treated with oral or intravenous iron, or with a medication for RLS/PLMS.

This study was approved by the institutional review board of the University of Montpellier, France. All patients provided written informed consent prior to participation.

### Polysomnography

All participants underwent a video‐PSG recording in the sleep laboratory from 11:00 pm (LIGHT‐OFF) to 7:00 am (LIGHT‐ON), followed by MSLT. Sleep was manually scored by experienced sleep experts, in 30‐sec epochs, based on the standard method.^16^ The following PSG parameters were collected: total sleep time, sleep efficiency, sleep latency, REM sleep latency, duration of wake after sleep onset, proportions of each stage of sleep, micro‐arousals, and apnea‐hypopnea indexes. Leg movements were simultaneously recorded by surface EMG from right and left anterior tibialis muscles. Periodic leg movements (PLM) were scored according to IRLSSG criteria, endorsed by World Association of Sleep Medicine.^17^ Patients with NT1 were further categorized according to their PLMS index below or above 15/h, while none of the controls had PLMS >15/h, by inclusion criteria. The mean sleep latency and number of sleep onset REM periods (SOREMP) were recorded on the MSLT. None of the participants took psychostimulants and anticataplectic medications or any other medication known to influence sleep or motor activity for at least 2 weeks prior to the PSG.

### Blood and CSF analysis

CSF samples were collected between 5:00 and 7:00 pm in all participants (*n* = 104), while blood samples were collected after overnight fasting between 7:00 and 7:30 am in 93 participants (63 patients with NT1 and 30 controls). After centrifugation, aliquots of CSF were immediately frozen and stored immediately at −80°C. CSF ORX‐A level was determined in duplicate using the I^125^‐radioimmunoassay (RIA) kit from Phoenix Pharmaceuticals, Inc (Belmont, CA, USA), according to the manufacturer's recommendations. Serum and CSF ferritin (Ser‐Ferr and CSF‐Ferr) levels were estimated by electrochemiluminiscence on Cobas 8000 e602© analyzer (Roche Diagnostics, Meylan, France) following the manufacturer's instructions.

### Statistical analysis

The characteristics of the study population were described using numbers and percentages for categorical variables and medians with range for continuous variables, as their distributions were mostly skewed according Shapiro–Wilk test. The relationships between demographic, clinical and biological variables and binary outcomes (First: presence vs. absence of RLS; Second: presence vs. absence of PLMS; and Third: NT1 patients vs. controls) were studied using logistic regression models. Non‐parametric tests were implemented to compare among NT1 patients, those with Ser‐Ferr levels below and above 50 and 75 ng/mL Spearman's rank order correlations were used to determine associations between continuous variables. Significant level was set at *P* < 0.05. Analyses were performed using SAS statistical software (version 9.4; SAS, Cary, NC).

## Results

### Serum and CSF ferritin levels in NT1 patients with and without RLS

Among the 66 NT1 patients, 20 (30.3%) patients had a comorbid moderate to severe RLS. These patients had a median age at onset of NT1 of 20.5 y.o. [8–39] and a median age at onset of RLS of 32.5 y.o. [13–55]. RLS started either at the same time or after the onset of narcolepsy for 86% of patients. One patient only reported a first‐degree familial history of RLS. Six NT1 patients with RLS, and six NT1 patients without RLS had sera ferritin levels below 50 ng/mL (Table 1).

**Table 1:** Clinical characteristics, PLM and ferritin levels in patients with NT1 with and without RLS.

Variable	NT1 without RLS *N* = 46		NT1 with RLS *N* = 20		*P*
	*n*	%	*n*	%	
Gender, women	15	32.61	7	35.00	0.85
Age, years^1^	39.00 (14.00; 80.00)		35.50 (15.00; 81.00)		0.46
BMI, kg/m^2^ ^1^	27.34 (19.49; 37.11)		25.67 (18.73; 34.61)		0.34
Hypnagogic/hypnopompic hallucinations, yes	32	74.42	16	80.00	0.63
Sleep paralysis, yes	25	58.14	13	65.00	0.60
Duration of evolution of NT1, years^1^	10.00 (1.00; 64.00)		5.00 (1.00; 69.00)		0.72
CSF ORX‐A levels, pg/mL^1^	10.50 (0.00; 101.00)		6.00 (0.00; 82.00)		0.42
PLM indexes					
Index PLMS, /h^1^	4.20 (0.00; 39.57)		3.24 (0.00; 161.90)		0.21
Index PLMS ≥15/h	12	26.09	6	30.00	0.74
Index PLMS associated with MA, /h^1^	1.30 (0.00; 21.23)		1.00 (0.00; 50.70)		0.24
Index of PLMS associated with MA ≥15/h	1	2.27	1	5.26	0.55
Index PLM wake, /h^1^	11.60 (0.00; 85.60)		9.57 (0.00; 105.60)		0.32
Index PLM wake ≥15/h	17	37.78	8	42.11	0.75
Ferritin levels					
Ser‐Ferr, ng/mL^1^	102.00 (12.00; 500.00)		95.00 (15.00; 281.00)		0.14
Ser‐Ferr, ng/mL ≤50	6	13.95	6	30.00	0.14
Ser‐Ferr, ng/mL ≤75	12	27.91	9	45.00	0.18
CSF‐Ferr, ng/mL^1^	6.82 (3.07; 14.89)		5.82 (3.00; 11.80)		0.27
CSF‐Ferr, ng/mL^2^					
<5.47	13	28.26	8	40.00	0.64
[5.47–7.73]	16	34.78	6	30.00	
≥7.73	17	36.96	6	30.00	

BMI, body mass index; CSF, cerebrospinal fluid; CSF‐Ferr, CSF ferritin levels; MA, microarousal; NT1, narcolepsy type 1; ORX‐A, orexin‐A levels; PLM, periodic leg movements; PLMS, periodic leg movements during sleep; RLS, restless legs syndrome; Ser‐Ferr, serum ferritin levels.

^1^ Continuous variables are expressed as median (minimal value; maximal value).

^2^ Tertiles of the NT1 sample (*n* = 66).

Compared to NT1 patients without RLS, those with RLS did not differ for age, gender and BMI, CSF ORX‐A levels, and for most clinical and electrophysiological characteristics: sleep‐related hallucinations, sleep paralysis, nocturnal sleep characteristics, indexes of PLMS, PLMS associated with microarousals and PLM in waketime, mean sleep latency, and number of SOREMP on MSLT (Table 1). No between‐group differences were found for Ser‐Ferr and CSF‐Ferr levels using either the median values or the tertiles of the sample (Table 1). The results remained unchanged when patients with Ser‐Ferr ≤50 or 75 ng/mL were excluded from the analysis.

### Serum and CSF ferritin levels in NT1 patients with and without PLMS

Among the 66 NT1 patients, 18 (27.3%) had a PLMS index ≥15/h, including six having comorbid moderate to severe RLS. In comparison to NT1 patients without PLMS (<15/h), those with PLMS were not different for demographic, clinical characteristics, and serum and CSF ferritin measurements using either the median values or the tertiles of the sample (Table 2). Results remained unchanged after excluding patients with Ser‐Ferr ≤50 or 75 ng/mL, comparing NT1 patients with RLS and/or PLMS (*n* = 32) to NT1 patients with no RLS and no PLMS (<15/h, *n* = 34) (median CSF‐Ferr levels 5.93 [3.00; 14.84] and 6.99 [3.07; 14.89] ng/mL respectively), or comparing NT1 patients with RLS and PLMS <15/h (*n* = 14) to NT1 patients without RLS and PLMS <15/h (*n* = 34).

**Table 2:** Clinical characteristics, PLM and ferritin levels in NT1 patients with and without PLMS.

Variable	NT1 with PLMS <15/h *N* = 48		with PLMS ≥15/h *N* = 18		*P*
	*n*	%	*n*	%	
Gender, women	17	35.42	5	27.78	0.56
Age, years^1^	38.00 (15.00; 76.00)		41.50 (14.00; 81.00)		0.13
BMI, kg/m^2^ ^1^	26.12 (18.73; 37.11)		27.01 (19.56; 31.30)		0.82
Hypnagogic/hypnopompic hallucinations, yes	34	72.34	14	87.50	
Sleep paralysis, yes	28	59.57	10	62.50	0.23
RLS, yes	14	29.17	6	33.33	0.74
Duration of evolution of NT1, years^1^	7.00 (1.00; 58.00)		14.50 (2.00; 69.00)		0.07
CSF ORX‐A levels, pg/mL^1^	11.00 (0.00; 101.00)		5.00 (0.00; 62.00)		0.20
PLM indexes					
Index PLMS, /h^1^	1.60 (0.00; 13.60)		27.01 (15.70; 161.90)		NA
Index PLMS associated with MA, /h^1^	0.50 (0.00; 8.40)		7.58 (0.90; 50.70)		<0.0001
Index of PLMS associated with MA ≥15/h	0	0.00	2	11.11	NA
Index PLM wake, /h^1^	8.94 (0.00; 65.20)		34.52 (1.70; 105.60)		0.0005
Index PLM wake ≥15/h	11	23.91	14	77.78	0.0003
Ferritin levels					
Ser‐Ferr, ng/mL^1^	99.00 (12.00; 500.00)		102.50 (28.00; 338.00)		0.83
Ser‐Ferr, ng/mL ≤50	10	22.22	2	11.11	0.32
Ser‐Ferr, ng/mL ≤75	15	33.33	6	33.33	0.99
CSF‐Ferr, ng/mL^1^	6.78 (3.00; 14.89)		5.93 (3.98; 14.84)		0.99
CSF‐Ferr, ng/mL^2^					
<5.47	15	31.25	6	33.33	0.37
[5.47–7.73]	14	29.17	8	44.44	
≥7.73	19	39.58	4	22.22	

BMI, body mass index; CSF, cerebrospinal fluid; CSF‐Ferr, CSF ferritin levels; MA, microarousal; NT1, narcolepsy type 1; ORX‐A, orexin‐A levels; PLM, periodic leg movements; PLMS, periodic leg movements during sleep; RLS, restless legs syndrome; Ser‐Ferr, serum ferritin levels; NA, test not applicable.

^1^ Continuous variables are expressed as median (minimal value; maximal value).

^2^ Tertiles of the NT1 sample (*n* = 66).

### Serum and CSF ferritin levels in NT1 patients and controls

We compared patients with NT1 with Ser‐Ferr ≤50 ng/mL to those above, and found no between‐group differences for comorbid RLS, PLMS index and for CSF‐Ferr levels (6.73 [3.00; 11.80] vs. 6.72 [3.61; 14.89]).

Compared to controls, NT1 patients were older, more frequently men, with higher BMI, and also higher ESS score and PLM indexes (Table 3). The control group had lower Ser‐Ferr levels than NT1 patients, with 50% of subjects having Ser‐Ferr ≤50 ng/mL (Table 3). CSF‐Ferr levels were also higher in patients with NT1 (Table 3, Model 0) but after adjustment for age, gender and Ser‐Ferr, this association became not significant (Table 3, Model 1). The results remained unchanged when also adjusted on BMI (i.e., a condition often associated with NT1), when CSF‐Ferr levels were divided in tertiles, after excluding subjects with Ser‐Ferr ≤50 ng/mL, or when comparing the subgroup of NT1 patients with RLS and/or PLMS with controls (data not shown).

**Table 3:** Clinical characteristics, PLM and ferritin levels in controls and NT1 patients.

Variable	Controls *N* = 38		NT1 patients *N* = 66		Model 0	Model 1
	*n*	%	*n*	%	*P*	*P*
Gender, women	26	68.42	22	33.33	0.0008	
Age, years^1^	22.50 (12.00; 61.00)		38.50 (14.00; 81.00)		0.0009	
BMI, kg/m^2^ ^1^	20.77 (16.45; 35.19)		26.12 (18.73; 37.11)		<0.0001	
ESS score^1^	15.00 (5.00 ;23.00)		20.00 (6.00 ;24.00)		0.001	
Duration of evolution of NT1, years^1^	–		10 (1.00; 69.00)		–	
PLM indexes						
Index PLMS, /h^1^	0.17 (0.00; 14.20)		4.10 (0.00; 161.90)		0.005	
Index PLMS ≥15/h	0	0	18	27.27	NA	
Index PLMS associated with MA, /h^1^	0.00 (0.00; 3.30)		1.30 (0.00; 50.70)		0.02	
Index of PLMS associated with MA ≥15/h	0	0	2	3.17	NA	
Index PLM wake, /h^1^	1.50 (0.00; 62.00)		11.55 (0.00; 105.60)		0.56	
Index PLM wake ≥15/h	7	18.42	25	39.06	0.87	
Ferritin levels						
Ser‐Ferr, ng/mL^1^	51.50 (6.00; 418.00)		100.00 (12.00; 500.00)		0.009	
Ser‐Ferr ≤50 ng/mL	15	50.00	12	19.05	0.003	
Ser‐Ferr ≤75 ng/mL	18	60.00	21	33.33	0.02	
CSF‐Ferr, ng/mL^1^	4.87 (2.09; 11.98)		6.64 (3.00; 14.89)		0.0008	0.09
CSF‐Ferr, ng/mL^2^						
<4.66	18	47.37	15	22.73	0.008	0.26
[4.66–6.89]	14	36.84	22	33.33		
≥6.89	6	15.79	29	43.94		

Model 0: crude association. Model 1: adjustment for age, gender, and serum ferritin levels. BMI, body mass index; CSF, cerebrospinal fluid; CSF‐Ferr, CSF ferritin levels; ESS, Epworth sleepiness scale; MA, microarousal; NT1, narcolepsy type 1; PLM, periodic leg movements; PLMS, periodic leg movements during sleep; RLS, restless legs syndrome; Ser‐Ferr, serum ferritin levels; NA, test not applicable.

^1^ Continuous variables are expressed as median (minimal value; maximal value).

^2^ Tertiles of the whole sample (*n* = 104).

In the whole population (patients and controls), CSF‐Ferr levels correlated positively with age (*r* = 0.59, *P* = 0.0001), Ser‐Ferr levels (*r* = 0.38, *P* = 0.0002), BMI (*r* = 0.3, *P* = 0.002), and negatively with ORX‐A levels (*r *= −0.26, *P* = 0.007). In patients with NT1, CSF‐Ferr levels also correlated with age (*r* = 0.51, *P* = 0.0001) and Ser‐Ferr levels (*r* = 0.26, *P* = 0.038). No association was found between PLMS index and CSF‐Ferr in the whole population, and in NT1 patients.

## Discussion

Our study found no CSF or serum ferritin level differences in ORX‐deficient narcolepsy comorbid with RLS or PLMS compared to those without RLS or PLMS, or controls. Our results strengthen a different pathophysiological mechanism underlying RLS comorbid with NT1 in comparison with primary RLS.

The pathophysiology of primary RLS is complex and remains incompletely resolved, but a brain iron deficiency has been documented in many neuropathological, biological, and brain‐imaging studies.^6^, ^18^, ^19^, ^20^, ^21^, ^22^, ^23^, ^24^ Recent clinical and population‐based studies did not report associations between RLS, PLMS, and serum ferritin levels.^6^, ^25^, ^26^, ^27^, ^28^ However, three major studies reported significant reduction of CSF ferritin concentrations in primary RLS, especially in early‐onset RLS (<45 years of age), with weaker correlations between CSF and serum ferritin levels in RLS than controls.^7^, ^8^, ^9^

The concept of RLS separated into two entities, primary or secondary to another condition, has been recently reconsidered, as a potential continuous spectrum with overlapping genetic and environmental risk factors.^3^, ^4^ Rare studies focused on the association between RLS and NT1.^5^, ^13^ Whether the pathophysiological mechanisms underlying RLS comorbid with NT1 differ than in primary RLS remains an open question. Previous studies showed that RLS in drug‐free patients with NT1 is often less severe, less familial, with less gender effect.^5^, ^13^ It is associated with less PLMS together with a lower periodicity index and a different nighttime distribution than primary RLS,^12^, ^13^ and it is not associated with low serum ferritin levels.^5^ To date, no CSF ferritin measurement was reported in NT1 patients comorbid with RLS. One study showed the absence of correlation between CSF ferritin levels and PLMS index in male and female separately with primary RLS^8^; however to our best knowledge, it has never been assessed in secondary RLS.

In this study, we included only drug‐free ORX‐deficient narcoleptic patients, with moderate to severe typical RLS symptoms diagnosed by a clinical interview, to avoid bias related to mild, atypical, and iatrogenic cases. As NT1 is an orphan disease and comorbid RLS is reported in ~15% of cases,^5^ we were able to include 20 untreated NT1 patients with RLS and compared them with other NT1 patients without RLS, not different for age, gender, and BMI. We found no differences on CSF or serum ferritin levels between groups. Herein, the median age at onset of RLS is 32.5 y.o. [13–55], thus almost all RLS patients may be phenotyped as early‐onset. Similar results were found when comparing NT1 patients with and without PLMS, or between NT1 patients with comorbid RLS and/or PLMS versus not. Results did not change when analysing the subgroup of NT1 patients with normal serum ferritin levels (>50 or >75 ng/mL) only. Moreover no differences were found for RLS, PLMS index and CSF ferritin levels between patients with NT1 with serum ferritin levels below or above 50 ng/mL.

Our control group with sleepiness complaint but without central hypersomnolence disorders, ORX‐A deficiency, RLS or PLMS, differs from the NT1 group for age, gender, BMI, and serum ferritin levels. After different adjustments for those potential confounders, all being associated with either CSF ferritin levels or NT1,^29^ no CSF ferritin level differences were found between groups. We found no association between CSF ferritin levels and PLMS index, neither in the whole study population, nor in NT1 population, confirming previous results in primary RLS.^29^ Unexpectedly, we found a mild negative correlation between CSF ferritin and ORX‐A levels in the whole population, but not in NT1 patients, certainly due to a floor effect. ORX neurons are centrally involved in motor control during wakefulness and sleep in humans, and ORX deficiency may cause a functional defect in the motor control leading to REM sleep behavior disorder (RBD), REM sleep without atonia, PLMS, and potentially RLS.^30^, ^31^, ^32^, ^33^ The pathophysiology of RLS in NT1 may be related to a common mechanism shared by the two disorders. This was already suggested to explain the occurrence of high PLMS in NT1.^11^ The best candidate is the dopamine system, a critical downstream mediator of ORX deficiency, and critically impaired in both conditions.^34^, ^35^, ^36^, ^37^ We recently showed a ^123^I‐metaiodobenzylguanidine cardiac uptake difference between RBD associated with NT1 and idiopathic RBD, providing a cardiac biomarker to differentiate those disorders.^38^ Altogether, these data further support the hypothesis of a functional defect in the motor control in NT1, probably specific to this disease and maybe linked to dysfunctions in ORX/dopaminergic system. We could thus speculate that the response to RLS/PLMS treatment, especially dopamine agonists and iron supplementation, would be different in the context of ORX‐deficient narcolepsy, probably less efficient, compared to patients with primary RLS.

We acknowledge some limitations in our study. The main limitation is the absence of patients with primary RLS and of healthy controls. As lumbar puncture is an invasive examination, we included as controls patients with daytime sleepiness complaint, for which a lumbar puncture had been performed as a part of diagnostic assessment, and finally without central hypersomnolence disorder. However, the CSF ferritin concentrations found in this study were in the same ranges as those previously reported in healthy subjects, confirming the normal values we obtained here in NT1 patients.^7^, ^9^ Patients with NT1 differed from the controls for age, gender, BMI, and serum ferritin levels; however we adjusted all the analyses for these potential confounding factors and finally found no significant between group differences for CSF ferritin levels, the main objective. The relative low number of NT1 patients with typical RLS could be questionable (20 patients compared to 46 without RLS), but this is a rare entity, in a rare sleep disease, and those patients were very well‐characterized in the present study, and all had a lumbar puncture. Moreover the previous CSF ferritin studies in humans, reporting differences between groups, included <25 subjects per group.^7^, ^9^ NT1 patients without RLS were chosen to be comparable for age, gender, and BMI with NT1 patients with RLS, allowing the interpretation of the results despite the rather small sample size. Post‐hoc power analysis were performed between the groups in our sample. With the means of CSF ferritin levels of 7.28 ng/mL in patients with NT1 without RLS, and 6.46 ng/mL in patients with NT1 with RLS and a global standard deviation of 2.79, 366 subjects would have been necessary to show significant between‐group differences with a power of 0.80 using a two‐sided two‐sample *t* test with a significance level of 0.05.

PLMS is a PSG finding that can be found in healthy subjects, associated with a number of medical, neurological, and even drug side effects, and that can be seen as an epi‐phenomenon due to advancing age. However the population study was not taking any medication known to influence sleep or motor activity for at least 2 weeks prior to the PSG, and groups of NT1 patients were – on purpose – comparable for age. It is also important to note that none of the participants of this study have been previously treated with oral or intravenous iron, which could have impacted the results of CSF ferritin stores. Finally, no common genetic variants associated with RLS were assessed in our NT1 population.

To conclude, our results demonstrate the absence of CSF ferritin deficiency in RLS or PLMS associated to NT1. This indicates normal brain iron levels in that condition, supporting different pathophysiological processes involved in RLS comorbid with NT1, and in primary RLS, remaining to be explored.

## Conflict of Interest

Y Dauvilliers received funds for seminars, board engagements and travel to conferences by UCB Pharma, Jazz, Theranexus, Flamel and Bioprojet. R Lopez received funds for speaking by UCB Pharma and Shire. L Barateau, S Chenini, E Evangelista, AL Rassu, AM Gorce‐Dupuy, M Lotierzo, and I Jaussent report no disclosures.
